# Regulation of vascular endothelial growth factor expression in human colon cancer by interleukin-1**β**

**DOI:** 10.1038/sj.bjc.6690553

**Published:** 1999-07

**Authors:** Y Akagi, W Liu, K Xie, B Zebrowski, R M Shaheen, L M Ellis

**Affiliations:** Departments of Cancer Biology and Surgical Oncology, The University of Texas MD Anderson Cancer Center, Houston, TX 77030, USA

**Keywords:** vascular endothelial growth factor, interleukin-1, colon cancer

## Abstract

Expression of vascular endothelial growth factor (VEGF), an important angiogenic factor in colon cancer, is tightly regulated by factors in the microenvironment. However, specific factors indigenous to the organ microenvironment of colon cancer growth that regulate VEGF expression in human colon cancer are not well defined. We investigated interleukin-1β (IL-1β) induction of VEGF expression in colon cancer cells and the mechanism by which this occurs. HT29 human colon cancer cells were treated with IL-1β for various periods. Induction of VEGF mRNA by IL-1β peaked at 24 h (> fivefold) and returned to baseline by 48 h. SW620 human colon cancer cells also reached a peak induction of VEGF mRNA 24 h after treatment with IL-1β. VEGF was induced at a dose range between 1 and 20 ng ml^−1^ of IL-1β. IL-1β induction of VEGF was also confirmed at the protein level. To examine the mechanism for VEGF induction by IL-1β, we transiently transfected VEGF promoter-reporter constructs into HT29 cells. IL-1β increased the activity of the VEGF promoter-reporter construct. Pretreatment of HT29 cells with dactinomycin abrogated the induction of VEGF mRNA by IL-1β. The half-life of VEGF mRNA was not prolonged by treatment with IL-1β. These findings suggest that IL-1β regulates VEGF expression in human colon cancer cells by increasing transcription of the VEGF gene. © 1999 Cancer Research Campaign


*British Journal of Cancer* (1999) **80**(10), 1506–1511
© 1999 Cancer Research Campaign
Article no. bjoc.1999.0553

### Regulation of vascular endothelial growth factor expression in human colon cancer by interleukin-1β

**Y Akagi[1], W Liu[1], K Xie[1], B Zebrowski[2], RM Shaheen[2] and LM Ellis[1,2]**

Departments of [1]Cancer Biology and [2]Surgical Oncology, The University of Texas MD Anderson Cancer Center, Houston, TX 77030, USA

**Summary** Expression of vascular endothelial growth factor (VEGF), an important angiogenic factor in colon cancer, is tightly regulated by factors in the microenvironment. However, specific factors indigenous to the organ microenvironment of colon cancer growth that regulate VEGF expression in human colon cancer are not well defined. We investigated interleukin-1β (IL-1β) induction of VEGF expression in colon cancer cells and the mechanism by which this occurs. HT29 human colon cancer cells were treated with IL-1β for various periods. Induction of VEGF mRNA by IL-1β peaked at 24 h (> fivefold) and returned to baseline by 48 h. SW620 human colon cancer cells also reached a peak induction of VEGF mRNA 24 h after treatment with IL-1β. VEGF was induced at a dose range between 1 and 20 ng $ml^{-1}$ of IL-1β. IL-1β induction of VEGF was also confirmed at the protein level. To examine the mechanism for VEGF induction by IL-1β, we transiently transfected VEGF promoter-reporter constructs into HT29 cells. IL-1β increased the activity of the VEGF promoter-reporter construct. Pretreatment of HT29 cells with dactinomycin abrogated the induction of VEGF mRNA by IL-1β. The half-life of VEGF mRNA was not prolonged by treatment with IL-1β. These findings suggest that IL-1β regulates VEGF expression in human colon cancer cells by increasing transcription of the VEGF gene.

**Keywords:** vascular endothelial growth factor; interleukin-1; colon cancer

Neovascularization is a critical requirement for tumour growth. The development of new blood vessels in tumours depends on the balance between stimulatory and inhibitory angiogenic factors released from the tumour cells or cells inhabiting the tumour microenvironment. Vascular endothelial growth factor (VEGF) has been implicated in driving the process of angiogenesis in a variety of tumours, including colon carcinoma (Takahashi et al, 1995). This factor, also known as vascular permeability factor or vasculotropin, was initially described as a protein that increases vascular permeability (Dvorak et al, 1979*a*, 1979*b*). Vascular permeability factor was later found to be identical to VEGF, an endothelial cell mitogen (Tischer et al, 1991). VEGF induces angiogenesis in numerous in vitro and in vivo assays (Dvorak et al, 1979a; Ellis et al, 1996). The biological activity of VEGF seems to be limited to endothelial cells. VEGF does not induce mitogenesis in nonendothelial cells (with rare exceptions), and antibodies to VEGF do not inhibit the growth of tumour cells in vitro (Kim et al, 1993).

VEGF is expressed in nearly all cell types, but many malignant tumour cells overexpress VEGF. Studies from our laboratory and others suggest that VEGF expression is intimately associated with inducing and maintaining the neovasculature of human colon cancers (Takahashi et al, 1995, 1997; Warren et al, 1995; Ellis et al, 1996, 1998). Although VEGF has received considerable study recently, the factors that regulate its production in colon cancer have not been fully elucidated. Hypoxia is one factor that has been shown to up-regulate VEGF in numerous tumour systems (Shweiki et al, 1995; Ellis et al, 1998). However, other factors are important in the up-regulation of VEGF expression (Rak et al, 1995; Gille et al, 1997; Ellis et al, 1998). VEGF is induced by several factors, and this ubiquitous sensitivity suggests that it plays an important role in the survival and growth of a tumour. In previous studies, we investigated the role of cytokines and growth factors indigenous to sites of colon cancer growth on VEGF induction. We initially found that insulin-like growth factor-I (IGF-I) and interleukin-1β (IL-1β) strongly induced VEGF expression in human colon cancer cells (Akagi et al, 1998). This previous report focused solely on the IGF-I induction of VEGF. In the present study, we further examine the role of IL-1β in inducing VEGF and the mechanism by which this occurs.

*Received 5 November 1998*
*Revised 5 January 1999*
*Accepted 26 January 1999*

*Correspondence to:* LM Ellis, Department of Surgical Oncology, Box 106, The University of Texas MD Anderson Cancer Center, 1515 Holcombe Blvd., Houston, TX 77030, USA

### MATERIALS AND METHODS

### Materials

Recombinant human IL-1β was purchased from R & D Systems, Inc. (Minneapolis, MN, USA). Dactinomycin (ActD) was purchased from Calbiochem-Novabiochem Corporation (La Jolla, CA, USA).

### Cell lines and culture conditions

The human colon cancer cell lines HT29 and SW620 were obtained from the American Type Culture Collection (Rockville, MD, USA). These cells were cultured and maintained in minimal essential medium (MEM) supplemented with 10% fetal bovine serum (FBS), 2 U $ml^{-1}$ penicillin–streptomycin, vitamins, 1 mM sodium pyruvate, 2 mM L-glutamine and non-essential amino acids at 37°C in 5% carbon dioxide and 95% air. All experiments were performed after the cells were grown to 90–95% confluence to avoid the effects of cell density on VEGF expression (Koura et al, 1996).

### IL-1β treatment of cells and time course of VEGF induction

To determine the time course of IL-1β induction of VEGF, HT29 cells were incubated in 5% serum-containing medium overnight and were then incubated in the presence or absence of IL-1β (10 ng $ml^{-1}$) for 1, 2, 4, 8, 24, or 48 h in serum-free medium. Total RNA was extracted from the cells as described below. VEGF mRNA expression was determined by Northern blot analysis. The supernatant of each sample was collected and centrifuged to remove debris, and stored at –70°C until assayed for protein level. We also examined the effect of IL-1β on VEGF expression in SW620 colon cancer cells to assure that the effect of IL-1β on VEGF induction was not due to a phenomenon unique to the HT29 cell line. The time at which VEGF expression peaked was used in subsequent studies.

### IL-1β dose–response of VEGF induction

To determine the dose–response of IL-1β induction of VEGF, HT29 cells were incubated in 5% serum-containing medium overnight and then in serum-free medium containing 0.1, 1, 5, 10, or 20 ng $ml^{-1}$ of IL-1-β for 24 h. Total RNA was extracted from the cells and Northern blots were done. Cells incubated in serum-free medium without IL-1β were used as controls at each time point. The supernatant of each sample was collected, centrifuged to remove debris and stored at –70°C.

### mRNA extraction and Northern blot analysis

Total RNA was extracted from cells by using Tri Reagent (Molecular Research Center, Inc., Cincinnati, OH, USA). Northern blot hybridization was performed as described previously (Koura et al, 1996). Briefly, total RNA (25 μg) was separated by electrophoresis in 1% denaturing formaldehyde-agarose gels. The RNA was transferred to the Hybond-N+ positively charged nylon membrane (Amersham Life Science, Arlington Heights, IL, USA) overnight by capillary elution and UV cross-linked at 120 000 mJ $cm^{-2}$ with a UV Stratalinker 1800 (Stratagene, La Jolla, CA, USA). After the blots were pre-hybridized for 3–4 h at 65°C in rapid hybridization buffer (Amersham), the membranes were hybridized overnight at 65°C with the cDNA probe for VEGF or glyceraldehyde 3-phosphate dehydrogenase (GADPH). The probed nylon membranes were then washed and exposed to radiographic film.

### cDNA probes

The cDNA probes used in this analysis were a human VEGF-specific 204-base-pair probe, a gift of Brygida Berse (Harvard Medical School, Boston, MA, USA) (Berse et al, 1992), and a GAPDH probe, purchased from the American Type Culture Collection. The VEGF probe identifies all alternatively spliced forms of its mRNA transcripts. Probes were purified by agarose gel electrophoresis by using the QIAEX Gel Extraction kit (QIAGEN, Inc., Chatworth, CA, USA). Each cDNA probe was radiolabelled with [$\alpha$-$^{32}$P] deoxyribonucleotide triphosphate by the random-priming technique with the Rediprime labelling system (Amersham).

### Determination of VEGF protein levels

The total protein concentration was quantified spectrophotometrically. The VEGF protein level in the supernatant was determined with an enzyme-linked immunosorbent assay (ELISA) kit according to the manufacturer’s instructions (R&D Systems, Minneapolis, MN, USA).

### VEGF promoter-reporter studies in response to IL-1β

The role of transcriptional regulation of VEGF by IL-1β was examined by using transient transfection techniques to examine the activity of the VEGF promoter-luciferase reporter construct. The following plasmids were used: pGL3-VEGF (containing the human VEGF promoter linked to the firefly luciferase reporter gene), pRLTK (an internal control plasmid containing the herpes simplex thymidine kinase promoter linked to a constitutively active Renilla luciferase reporter gene), and pGL3 (plasmid vector alone as a negative control) (Akagi et al, 1998). HT29 cells ($5 \times 10^6$) were seeded and grown to 60–70% confluence. pRLTK and pGL3-VEGF (5 mg:20 mg) were co-transfected into cells by using the LipoFectin method (Life Science, Grand Island, NY, USA) as outlined by the manufacturers. pRLTK and pGL3 were co-transfected as a negative control. After cells were incubated in the transfection medium for 20 h, the medium was changed to standard medium and incubated for another 12 h. Cells were then incubated in the presence or absence of IL-1β or cobalt chloride ($CoCl_2$) (200 μM) for 24 h (the period determined to afford the greatest induction of VEGF mRNA). Cells were harvested with passive lysis buffer (Dual-Luciferase Reporter Assay System; Promega, Madison, WI, USA), and luciferase activity was determined with a single sample luminometer as outlined in the manufacturer’s protocol. $CoCl_2$ was used as a positive control because of its ability to mimic the hypoxic response (Minchenko et al, 1994).

### Effect of IL-1β on VEGF transcriptional activity

To confirm that the increase in VEGF mRNA in colon cancer cells was due to an increase in transcription, transcriptional activity was blocked with ActD before the cells were treated with IL-1β. HT29 cells, incubated in 5% serum MEM overnight, were incubated in the presence or absence of ActD (1 μg $ml^{-1}$) 2 h before their exposure to IL-1β (optimal dosing to avoid cell death was determined in preliminary experiments) in serum-free medium. Total RNA was extracted from the cells after 24 h, and Northern blot analysis was done. Control cells were treated with ActD without IL-1β.

### VEGF mRNA half-life after induction by IL-1β

To determine the effect of IL-1β on VEGF mRNA stability, HT29 cells were incubated in the presence or absence of IL-1β for 24 h. Further transcription in cells was then blocked by the addition of ActD (final concentration of 1 μg $ml^{-1}$). Total RNA was extracted from the cells 0, 0.25, 0.5, 1, 2 and 4 h after the addition of ActD, and Northern blot analysis was done for VEGF mRNA expression. VEGF mRNA expression at each time point was compared to the

© 1999 Cancer Research Campaign

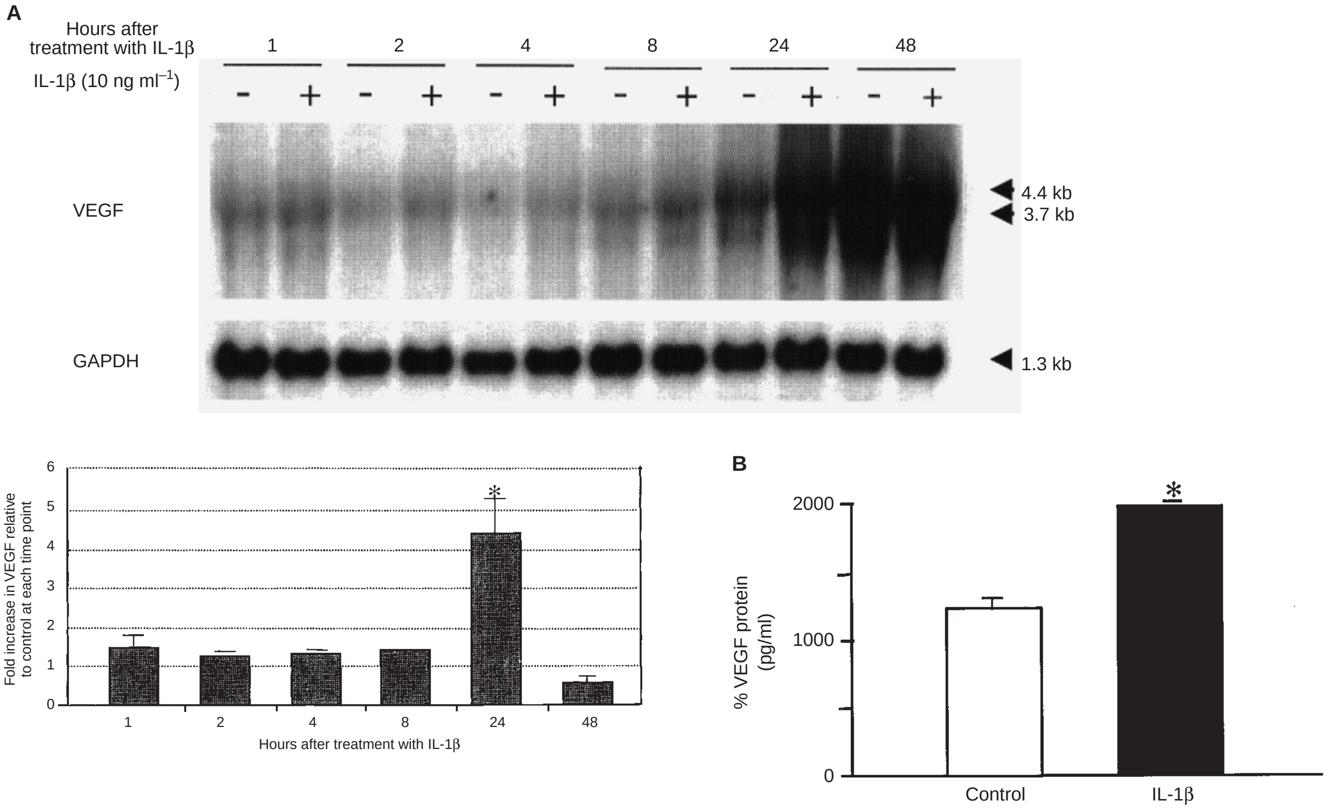

**Figure 1** Time course of IL-1β induction of VEGF in human colon cancer cells. (**A**) HT29 cells were treated with IL-1β in serum-free medium for the indicated times, total RNA was extracted, and Northern blot analyses was done for expression of VEGF mRNA. A representative Northern blot is shown in the upper panel. For each time point, untreated cells were used as a control as previously described (Akagi, 1998 #408). GAPDH mRNA transcripts were used as an internal control to correct for loading differences. In the lower panel, a bar graph depicts relative increases in VEGF expression by densitometric analysis in experiments repeated for each time point. On average, IL-1β induced a greater than fourfold increase in VEGF mRNA expression in HT29 cells at 24 h. *Denotes significant difference from untreated cells at each time point by unpaired Student's *t*-test. (**B**) VEGF protein level was measured in the conditioned medium by ELISA 24 h after treatment with IL-1β. VEGF protein secreted from cells treated with IL-1β was 80% greater than the control control value (total RNA extracted from cells before ActD treatment was arbitrarily defined as 100%). The half-life of VEGF mRNA was determined by plotting relative VEGF mRNA expression levels on a semilogarithmic axis versus time (Cricket Software, Malvern, PA, USA).

### Densitometric quantification

The software program Image Quant (Molecular Dynamics, Sunnyvale, CA, USA) was used to quantify the VEGF mRNA expression and GAPDH mRNA transcripts in the linear range of the film. GAPDH mRNA was used as an internal control for loading. Statistical analysis was done using InStat software for Macintosh (GraphPad Software, San Diego, CA, USA).

### RESULTS

### Time course of IL-1β induction of VEGF mRNA and protein in human colon cancer cells

VEGF mRNA expression in HT29 cells had increased more than five times the baseline level at 24 h after treatment with IL-1β (Figure 1). However, control cells that were grown in serum-free medium without the addition of IL-1β also exhibited an increase in VEGF mRNA expression, which was most pronounced at 48 h. We have observed this effect before, when we demonstrated that serum starvation increases VEGF mRNA between 24 and 48 h after incubation in serum-free medium (Akagi et al, 1998). Therefore, after densitometric analysis, we corrected for the increase in VEGF expression due to serum starvation by subtracting it from the increase in VEGF mRNA expression in the cells treated with IL-1β (Akagi et al, 1998). In SW620 cells, the peak induction of VEGF by IL-1β also occurred at 24 h, and the extent of the increase was similar to that of HT29 cells (data not shown).

To determine whether the increase in VEGF mRNA expression induced by IL-1β was associated with an increase in VEGF protein, we measured VEGF protein levels in the supernatants by ELISA (Figure 1B). In cells treated with IL-1β, VEGF protein levels increased 80% in the supernatants compared to control cells.

### Dose–response of VEGF mRNA and protein secretion induced by IL-1β in HT29 cells

We also determined the dose–response relationship of VEGF induction by IL-β by incubating HT29 cells in increasing concentrations of IL-1β for 24 h. The increase in VEGF expression at doses ranging from 1 ng ml$^{-1}$ to 20 ng ml$^{-1}$ followed a plateau pattern (Figure 2A). VEGF protein levels also increased at doses greater than 1 ng ml$^{-1}$, but not so much as the mRNA (Figure 2B).

 *© 1999 Cancer Research Campaign*

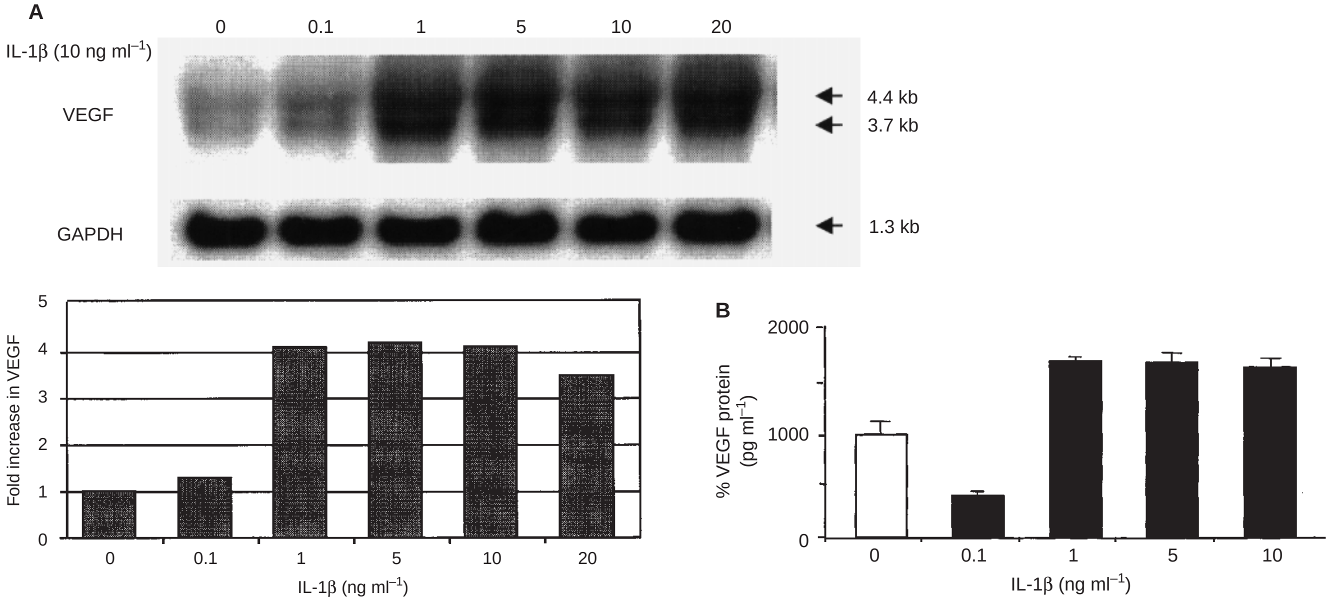

**Figure 2** Effect of increasing concentrations of IL-1β on VEGF expression in HT29 cells. (**A**) HT29 cells were incubated with the indicated concentrations of IL-1β (0.1, 1, 5, 10 ng ml$^{-1}$) for 24 h (the peak of VEGF expression in HT29 cells). Northern blot analyses were done to determine VEGF mRNA expression. IL-1β induction of VEGF reached a plateau between 1 and 20 ng ml$^{-1}$ with increases 3–4 times those of the control. (**B**) VEGF protein levels in supernatants from cells treated with 1 to 20 ngml$^{-1}$ of IL-1β also were 80% greater than the control

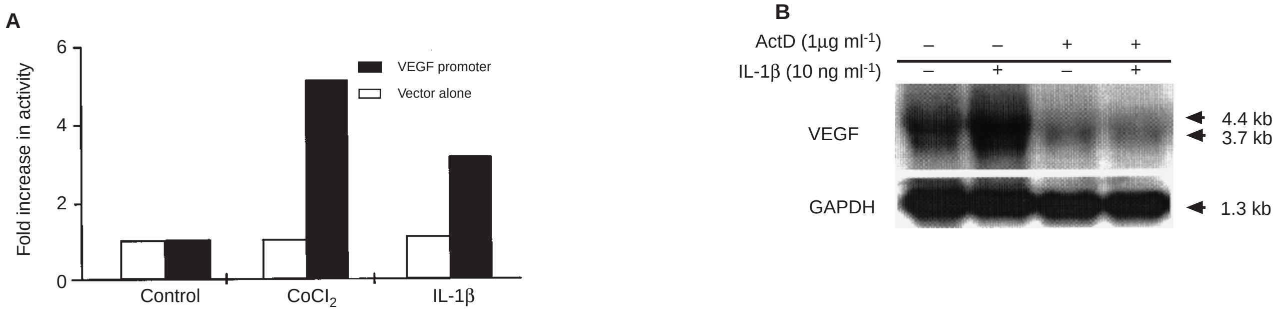

**Figure 3** Induction of VEGF transcription by IL-1β. (**A**) HT29 cells were co-transfected with pGL3-VEGF (VEGF promoter-luciferase reporter construct) and pRLTK (to control for transfection efficiency), with the negative control being transfection with pGL3 and pRLTK. Twenty-four hours after transient transfection, cells were treated with IL-1β (10 ng ml$^{-1}$), $CoCl_2$ (200 mM; positive control), or serum-free medium (negative control) for 24 h. Luciferase activity was determined, and relative promoter activity was displayed graphically. IL-1β increased the activity of the VEGF promoter constructs ~ threefold. (**B**) HT29 cells were treated with ActD (1 μg ml$^{-1}$) for 2 h to block further transcription, after which cells were treated with IL-1β for 24 h, and RNA was extracted and northern blots were done. Cells treated with IL-1β but not ActD were used as a positive control. Pretreatment with ActD blocked IL-1β induction of VEGF mRNA

### IL-1β increased VEGF mRNA expression by an increase in transcriptional activity

To determine the mechanism by which IL-1β induces VEGF, transient transfections were done with VEGF promoter-reporter constructs in HT29 cells. Control cells transfected with pGL3 (plasmid vector alone) and pRLTK demonstrated no increase in promoter activity (Figure 3A). Cells transfected with pGL3-VEGF (promoter-reporter construct) and pRLTK and treated with $CoCl_2$ (positive control) demonstrated a more than 500% increase in activity. Cells transfected with pGL3-VEGF and pRLTK and treated with IL-1β similarly demonstrated an almost 300% increase in activity.

To determine that the mechanism by which IL-1β induced VEGF mRNA expression occurred at a transcriptional level, transcription in HT29 cells was blocked with ActD before the addition of IL-1β. This transcription blockade completely inhibited the induction of VEGF mRNA expression (Figure 3B). These results suggest that IL-1β induction of VEGF is regulated by an increase in transcription of the gene.

### IL-1β did not alter the stability of VEGF mRNA

To further explore the mechanism by which IL-1β enhanced the expression of VEGF mRNA, we investigated the stability of VEGF mRNA by examining its half-life. The half-life of VEGF mRNA treated with IL-1β was similar to that of cells not exposed to IL-1β, demonstrating that the half-life of VEGF mRNA was not prolonged by treatment with IL-1β (Figure 4).

### DISCUSSION

Several laboratories have demonstrated that the degree of neovascularization in primary colon cancer specimens correlates with tumour aggressiveness (Frank et al, 1995; Takahashi et al, 1995, 1997). Previous studies from our laboratory have demonstrated an

*© 1999 Cancer Research Campaign* 

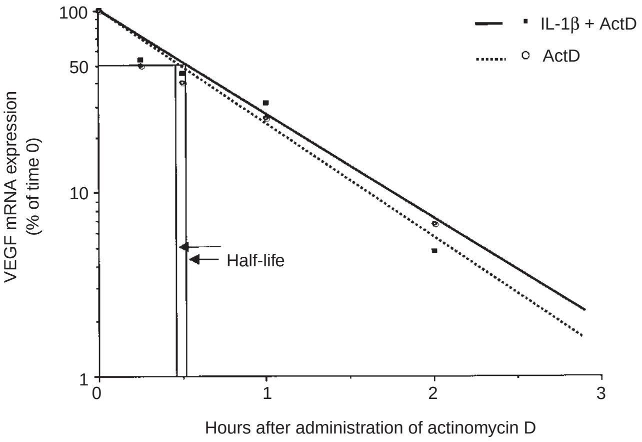

**Figure 4** Effect of IL-1β on VEGF mRNA half-life. HT29 cells were incubated in the presence or absence of IL-1β for 24 h before being exposed to ActD (1 μg ml$^{-1}$). Total RNA was extracted from cells at the indicated times after the addition of ActD, and northern blots were done to determine VEGF mRNA expression. VEGF mRNA expression was calculated relative to control, and the half-life was determined by plotting representative relative VEGF expression values on a semilogarithmic scale. IL-1β did not affect the half-life of VEGF mRNA

association between VEGF expression and vessel count in colon cancer (Takahashi et al, 1995, 1997). Other studies have also demonstrated the importance of VEGF in the growth and metastasis of human colon cancer; for example, giving neutralizing VEGF antibodies to mice bearing human colon cancer xenografts decreases tumour growth and inhibits metastasis (Warren et al, 1995). Thus VEGF seems to be an important factor in determining the angiogenic phenotype in human colon cancers.

In experimental tumour models, increased expression of VEGF has been associated with increased tumour growth. Transfection of VEGF into a breast carcinoma cell line increased in vivo tumour growth and vessel density (Zhang et al, 1995). In a similar study, overexpression of VEGF by a melanoma cell line increased tumour growth, angiogenesis and experimental metastasis (Claffey et al, 1996). In another study, the growth and vascularity of a rat glioma cell line in nude mice decreased after transfection with an antisense VEGF construct (Saleh et al, 1996).

Neutralizing antibodies to VEGF decrease tumour growth in mice bearing tumours that express VEGF (e.g. glioblastoma, rhabdomyosarcoma and leiomyosarcoma) (Kim et al, 1993). Tumour cell proliferation in vitro was not affected by the antibodies, suggesting that the decrease in tumour growth in vivo was due to the VEGF antibodies' anti-angiogenic effect. More recently, antibodies to VEGF decreased tumour growth in vivo in an experimental model of liver metastasis from colon cancer (Warren et al, 1995).

VEGF participates in numerous physiologic processes and has been detected in nearly all cells in the body. Therefore, factors that up-regulate its expression may contribute to angiogenesis and a more aggressive tumour phenotype. Hypoxia, the best-characterized mediator of VEGF induction, increases VEGF expression within 3–6 h; subsequent normalization of oxygen tension causes VEGF mRNA to return to baseline levels (Ikeda et al, 1995). Hypoxic induction of VEGF may be regulated by an increase in the transcription or stabilization of the mRNA (Ikeda et al, 1995; Levy et al, 1995, 1996). However, hypoxia is but one of a large number of factors that induce VEGF expression. Several cytokines and growth factors are known to affect VEGF expression as well. Factors shown to increase VEGF expression in tumour cells include tumour necrosis factor, transforming growth factor-α, epidermal growth factor, IGF-I and PDGF-BB (Akagi et al, 1998; Tsai et al, 1995; Ryuto et al, 1996). However, not all of these factors increase VEGF expression in all tumour systems, i.e. the factors involved in the regulation of VEGF may depend on the tumour system under study. In addition, it is not clear whether these factors affect common signal transduction pathways or multiple pathways in the regulation of VEGF expression.

IL-1β is known to stimulate the proliferation of vascular smooth muscle cells and is involved in modifying a number of vascular functions by inducing autocrine production of chemotactic cytokines on endothelial cells, including IL-1β itself (Libby et al, 1988; Mantovani and Dejana, 1989; Mantovani et al, 1992). Moreover, IL-1β has been shown to enhance the production of IL-8 protein, which has been shown to induce angiogenesis in melanoma cells (Koch et al, 1992; Strieter et al, 1992; Gutman et al, 1995). These observations suggest that IL-1β may alter the functional properties of vascular cells, including the induction of angiogenesis. Vascular-biology studies have shown that IL-1β increase VEGF mRNA in rat aortic smooth muscle cells in a time- and dose-dependent manner (Li et al, 1995). This induction of VEGF expression was found to be due to an increase in transcription as well as an increase in the stability of its mRNA. However, the role of IL-1β on the induction of VEGF in tumours, specifically human colon cancer, is undefined.

We have demonstrated that IL-1β induced VEGF mRNA expression in two human colon cancer cell lines. VEGF mRNA induction by IL-1β peaked at 24 h, and no demonstrable dose–response was found above an initial threshold concentration. The increase in VEGF expression was due to an increase in transcription of the VEGF gene, as shown by two separate investigative strategies. However, the half-life of VEGF mRNA induced by IL-1β was not prolonged. The induction of VEGF transcription by IL-1β treatment is consistent with findings in other cell lines (Li et al, 1995). The observation that the mRNA half-life was not prolonged is consistent with the effect of other cytokines and growth factors on VEGF induction (Akagi et al, 1998). Others also have shown that IL-1β induces expression of another member of the VEGF family, VEGF-C, in lung fibroblasts by an increase in transcription alone (Ristimaki et al, 1998).

Little is known about the transcription factors that are activated by treatment with IL-1β. IL-1β has been shown to mediate the transcriptional activation of IL-2 through AP-1 sites, and the 5′ flanking region of the VEGF gene contains several AP-1 binding sites (Muegge et al, 1989; Tischer et al, 1991). It is therefore possible that IL-1β increases VEGF transcription by activating pathways that lead to AP-1 activation and binding to the VEGF promoter.

IL-1β is an inflammatory cytokine present in activated immune cells. Solid tumours, including colon cancer, are infiltrated by numerous immune effector cells, including macrophages and lymphocytes, through the expression of platelet-derived endothelial cell growth factor (Takahashi et al, 1996). This infiltration of immune effector cells occurs in both primary and metastatic cancer. We have shown elsewhere that infiltrating cells may contribute to the angiogenesis of human colon cancer (Takahashi et al, 1996). IL-1β induction of VEGF in human colon cancer cells could be another mechanism by which infiltrating cells contribute to tumour angiogenesis.

© 1999 Cancer Research Campaign

### ACKNOWLEDGEMENTS

This work was supported in part by American Cancer Society Career Development Award 94-21, the Gillson Longenbaugh Foundation, and the Charlotte Geyer Foundation (all to LME), National Institutes of Health T-32 grant CA09599 (BZ, RS) and an American Cancer Society Award (BZ). The authors thank Christine Wogan for editorial assistance.

### REFERENCES

Akagi Y, Liu W, Xie K, Zebrowski B and Ellis LM (1998) Regulation of vascular endothelial growth factor expression in human colon cancer by insulin-like growth factor-I. *Cancer Res* **58**: 4008–4014

Berse B, Brown LF, vanDeWater L, Dvorak HA and Senger DR (1992) Vascular permeability factor (vascular endothelial growth factor) is expressed differentially in normal tissues, macrophages, and tumors. *Mol Biol Cell* **3**: 211–220

Claffey KP, Brown LF, del Aguila LF, Tognazzi K, Yeo KT, Manseau EJ and Dvorak HF (1996) Expression of vascular permeability factor/vascular endothelial growth factor by melanoma cells increases tumor growth, angiogenesis, and experimental metastasis. *Cancer Res* **56**: 172–181

Dvorak HF, Dvorak AM, Manseau EJ, Wiberg L and Churchill WH (1979*a*) Fibrin-gel investment associated with line 1 and line 10 solid tumor growth, angiogenesis, and fibroplasia in guinea pigs. Role of cellular immunity, myofibroblasts, microvascular damage, and infarction in line 1 tumor regression. *J Natl Cancer Inst* **62**: 1459–1472

Dvorak HF, Orenstein NS, Carvalho AC, Churchill WH and Dvorak AM (1979*b*) Induction of a fibrin-gel investment: an early event in line 10 hepatocarcinoma growth mediated by tumor-secreted products. *J Immunol* **122**: 167–174

Ellis LM, Liu W and Wilson M (1996) Down-regulation of vascular endothelial growth factor in human colon carcinoma cell lines by antisense transfection decreases endothelial cell proliferation. *Surgery* **120**: 871–878

Ellis LM, Staley CA, Liu W, Fleming RYD, Parikh N, Bucana CD and Gallick GE (1998) Down-regulation of vascular endothelial growth factor in a human colon carcinoma cell line transfected with an antisense expression vector specific for c-src. *J Biol Chem* **273**: 1052–1057

Frank RE, Saclarides TJ, Leurgans S, Speziale NJ, Drab EA and Rubin DB (1995) Tumor angiogenesis as a predictor of recurrence and survival in patients with node-negative colon cancer [see comments]. *Ann Surg* **222**: 695–699

Gille J, Swerlick RA and Caughman SW (1997) Transforming growth factor-alpha-induced transcriptional activation of the vascular permeability factor (VPF/VEGF) gene requires AP-2-dependent DNA binding and transactivation. *EMBO J* **16**: 750–759

Gutman M, Singh RK, Xie K, Bucana CD and Fidler IJ (1995) Regulation of interleukin-8 expression in human melanoma cells by the organ environment. *Cancer Res* **55**: 2470–2475

Ikeda E, Achen MG, Breier G and Risau W (1995) Hypoxia-induced transcriptional activation and increased mRNA stability of vascular endothelial growth factor in C6 glioma cells. *J Biol Chem* **34**: 19761–19768

Kim KJ, Li B, Winer J, Armanini M, Gillett N, Phillips HS and Ferrara N (1993) Inhibition of vascular endothelial growth factor-induced angiogenesis suppresses tumour growth in vivo. *Nature* **362**: 841–844

Koch AE, Polverini PJ, Kunkel SL, Harlow LA, DiPietro LA, Elner VM, Elner SG and Strieter RM (1992) Interleukin-8 as a macrophage-derived mediator of angiogenesis [see comments]. *Science* **258**: 1798–1801

Koura AN, Radinsky R, Kitadai Y, Takahashi Y, Liu W, Singh R and Ellis LM (1996) Regulation of vascular endothelial growth factor expression in human colon carcinoma cells by cell density. *Cancer Res* **56**: 3891–3894

Levy AP, Levy NS, Wegner S and Goldberg MA (1995) Transcriptional regulation of the rat vascular endothelial growth factor gene by hypoxia. *J Biol Chem* **270**: 13333–13340

Levy AP, Levy NS and Goldberg MA (1996) Post-transcriptional regulation of vascular endothelial growth factor by hypoxia. *J Biol Chem* **271**: 2746–2753

Li J, Perrella MA, Tsai JC, Yet SF, Hsieh CM, Yoshizumi M, Patterson C, Endege WO, Zhou F and Lee ME (1995) Induction of vascular endothelial growth factor gene expression by interleukin-1b in rat aortic smooth muscle cells. *J Biol Chem* **270**: 308–312

Libby P, Warner SJ and Friedman GB (1988) Interleukin 1: a mitogen for human vascular smooth muscle cells that induces the release of growth-inhibitory prostanoids. *J Clin Invest* **81**: 487–498

Mantovani A, Bussolino F and Dejana E (1992) Cytokine regulation of endothelial cell function. *Faseb J* **6**: 2591–2599

Mantovani A and Dejana E (1989) Cytokines as communication signals between leukocytes and endothelial cells. *Immunol Today* **10**: 370–375

Minchenko A, Bauer T, Salceda S and Caro J (1994) Hypoxic stimulation of vascular endothelial growth factor expression in vitro and in vivo. *Lab Invest* **71**: 374–379

Muegge K, Williams TM, Kant J, Karin M, Chiu R, Schmidt A, Siebenlist U, Young HA and Durum SK (1989) Interleukin-1 costimulatory activity on the interleukin-2 promoter via AP-1. *Science* **246**: 249–251

Rak J, Mitsuhashi Y, Bayko L, Filmus J, Shirasawa S, Sasazuki T and Kerbel RS (1995) Mutant ras oncogenes upregulate VEGF/VPF expression: implications for induction and inhibition of tumor angiogenesis. *Cancer Res* **55**: 4575–4580

Ristimaki A, Narko K, Enholm B, Joukov V, Alitalo K (1998) Proinflammatory cytokines regulate expression of the lymphatic endothelial mitogen vascular endothelial growth factor-c. *J Biol Chem* **273**: 8413–8418

Ryuto M, Ono M, Izumi H, Yoshida S, Weich HA, Kohno K and Kuwano M (1996) Induction of vascular endothelial growth factor by tumor necrosis factor alpha in human glioma cells. Possible roles of SP-1. *J Biol Chem* **271**: 28220–28228

Saleh M, Stacker SA and Wilks AF (1996) Inhibition of growth of C6 glioma cells in vivo by expression of antisense vascular endothelial growth factor sequence. *Cancer Res* **56**: 393–401

Shweiki D, Neeman M, Itin A and Keshet E (1995) Induction of vascular endothelial growth factor expression by hypoxia and by glucose deficiency in multicell spheroids: implications for tumor angiogenesis. *Proc Natl Acad Sci USA* **92**: 768–772

Strieter RM, Kunkel SL, Elner VM, Martonyi CL, Koch AE, Polverini PJ and Elner SG (1992) Interleukin-8. A corneal factor that induces neovascularization. *Am J Pathol* **141**: 1279–1284

Takahashi Y, Bucana CD, Liu W, Yoneda J, Kitadai Y, Cleary KR and Ellis LM (1996) Platelet derived endothelial cell growth factor in human colon cancer angiogenesis: role of infiltrating cells. *J Natl Cancer Inst* **88**: 1146–1151

Takahashi Y, Kitadai Y, Bucana CD, Cleary KR and Ellis LM (1995) Expression of vascular endothelial growth factor and its receptor, KDR, correlates with vascularity, metastasis, and proliferation of human colon cancer. *Cancer Res* **55**: 3964–3968

Takahashi Y, Tucker SL, Kitadai Y, Koura AN, Bucana CD, Cleary KR and Ellis LM (1997) Vessel counts and VEGF expression as prognostic factors in node-negative colon cancer. *Arch Surg* **132**: 541–546

Tischer E, Mitchell R, Hartman T, Silva S, Gospodarowicz D, Fiddes JC and Abraham JA (1991) The human gene for vascular endothelial growth factor. Multiple protein forms are encoded through alternate exon splicing. *J Biol Chem* **266**: 11947–11954

Tsai JC, Goldman CK and Gillespie GY (1995) Vascular endothelial growth factor in human glioma cell lines: induced secretion by EGF, PDGF-BB, and bFGF. *J Neurosurg* **82**: 864–873

Warren RS, Yuan H, Matli MR, Gillett NA and Ferrara N (1995) Regulation by vascular endothelial growth factor of human colon cancer tumorigenesis in a mouse model of experimental liver metastasis. *J Clin Invest* **95**: 1789–1797

Zhang HT, Craft P, Scott PE, Siche M, Weich HA, Harris AL and Bicknell R (1995) Enhancement of tumor growth and vascular density by transfection of vascular endothelial cell growth factor into MCF-7 human breast carcinoma cells. *J Natl Cancer Inst* **87**: 213–219.

© 1999 Cancer Research Campaign
